# Sensitivity to electrical stimulation of human immunodeficiency virus type 1 and MAGIC-5 cells

**DOI:** 10.1186/2191-0855-1-23

**Published:** 2011-08-08

**Authors:** Etsuko Kumagai, Masato Tominaga, Shinji Harada

**Affiliations:** 1 Ex-Department of Biomedical Laboratory Sciences, Faculty of Life Sciences, Kumamoto University, 4-24-1, Kuhonji, Kumamoto 862-0976, Japan; 2 Graduate School of Science and Technology, Kumamoto University, 2-39-1 Kurokami, Kumamoto 860-8555, Japan; 3 Department of Medical Virology, Faculty of Life Sciences, Kumamoto University, 1-1-1 Honjo, Kumamoto 860-8556, Japan

**Keywords:** HIV-1 infectivity, electrical stimulation, indium-tin oxide, poly-L-lysine

## Abstract

To determine the sensitivities to low electrical potential of human immunodeficiency virus type 1 (HIV-1) and its target cells, HIV-1 and MAGIC-5 cells were directly stimulated with a constant direct current potential of 1.0 V (vs. Ag/AgCl). HIV-1 was incubated for 3 h at 37°C on a poly-L-lysine-coated indium-tin oxide electrode, and then stimulated by an electrical potential. MAGIC-5 cells were seeded onto the electrically stimulated HIV-1 and cultured for 3 days at 37°C. HIV-1-infected cells were measured by multinuclear activation via a galactosidase indicator assay. MAGIC-5 cells were also stimulated by an electrical potential of 1.0 V; cell damage, proliferation and apoptosis were evaluated by trypan blue staining, cell counting and *in situ *apoptosis detection, respectively. HIV-1 was found to be damaged to a greater extent by electrical stimulation than the cells. In particular, after application of a 1.0-V potential for 3 min, HIV-1_LAI _and HIV-1_KMT _infection were inhibited by about 90%, but changes in cell damage, proliferation and apoptosis were virtually undetectable. These results suggested that HIV-1 is significantly more susceptible to low electrical potential than cells. This finding could form the basis of a novel therapeutic strategy against HIV-1 infection.

## Introduction

Infection with human immunodeficiency virus type 1 (HIV-1), the causative agent of acquired immunodeficiency syndrome (AIDS), leads to depressed cellular immunity and can result in co-infection with opportunistic pathogens and severe disease (Gottlie et al. 1981; Masur et al. 1981; Aboulafia 2000; Picker et al. 2006). The available treatments for HIV infection include anti-HIV-1 therapy that inhibits the growth of the virus and prevents or reduces infection caused by various opportunistic pathogens. By using highly active anti-retroviral therapy, the morbidity and mortality rates in HIV-1-infected individuals have dramatically declined (Hogg et al. 1997; Palella et al. 1998). However, such intensive anti-retroviral therapy has several drawbacks, including drug side-effects, the complexity of the therapeutic regimen and the appearance of resistant HIV-1 strains (Carr et al. 1998; Colgrove et al. 1998; Samati et al. 2002). In 2010, human monoclonal antibodies, neutralizing over 90% of circulating HIV-1 isolates, were identified (Wu et al. 2010; Zhou et al. 2010). The therapeutic use of multiple broadly neutralizing human monoclonal antibodies to HIV-1 would therefore be expected to block HIV-1 infection. However, effective vaccines based on this strategy are yet to be developed and much interest remains in developing therapies based on novel principles.

The effects of electrical stimulation on living cells have been extensively studied since the 1970s, and changes in cellular responses have been observed. In fact, cell membrane damage (Tominaga et al. 2007), the regulation of cell proliferation (Kojima et al. 1991; Yaoita et al. 1990), gene expression of nerve growth factor (Koyama et al. 1996; Koyama et al. 1997) and neural and osteogenic differentiation (Kimura et al. 1998; Mie et al. 2003), have been reported in response to low potential loading. Furthermore, the combined effect of low potential stimulation and cisplatin administration caused cell death in HeLa cells (Manabe et al. 2004).

We are the only investigators to report of an electrical stimulation method as a means of protection against viral infection. One of our main findings was that the sensitivity to electrical stimulation was greater in chronically infected HIV-1_LAI _(a T-cell-tropic strain of HIV) HeLa cells compared with uninfected HeLa cells (Tominaga et al. 2003; Kumagai et al. 2004). Another finding has indicated that reactive oxygen species (ROS) induced by electrical stimulation play a role in inhibition of HIV-1 infection (Kumagai et al. 2007). Despite many reports regarding the effects of electrical stimulation on cells, no previous studies have examined the effects of electrical stimulation on viruses. It is possible that if HIV-1 has high sensitivity to a low-electric potential compared with host cells, HIV-1 could be specifically inactivated by this treatment without any damage to host cells.

Poly-L-lysine (PLL) is a nonspecific attachment factor for cells, useful in promoting cell adsorption to solid substrates (Yavin et al. 1974; McKeehan et al. 1984; Atashi et al. 2009). Because PLL is a cationic agent, it enhances electrostatic interaction between negatively-charged ions of the cell membrane and culture surface. Coating indium-tin oxide (ITO) electrode surfaces with PLL increases the number of positively charged ions on the ITO electrode surface, which might allow adsorption of virus on the ITO electrode.

To determine the low-electric potential sensitivity of HIV-1 and cells, HIV-1 and MAGIC-5 cells were adsorbed onto a PLL-coated ITO electrode and directly stimulated with a constant direct current (d.c.) potential of 1.0 V (vs. Ag/AgCl). HIV-1 infectivity, cell damage, cell proliferation and the numbers of apoptotic cells were then examined to determine the sensitivities of HIV-1 and cells to electrical stimulation.

## Materials and methods

### Preparation of virus

HIV-1_LAI_, a T-tropic HIV, was propagated in persistently infected Molt4 cells as previously described (Koyanagi et al. 1986), and HIV-1_KMT_, a dual-tropic HIV, was propagated in persistently infected CEM cells as previously described (Morikita et al. 1997). Cell-free viruses were obtained by filtration of the cell supernatants through 0.45-μm filters (Millipore, Bedford, UK). Viruses were then aliquoted and stored at -80°C prior to use. The concentration of HIV-1_LAI _and HIV-1_KMT _core p24 proteins were 211 ng/ml and 157 ng/ml, respectively.

### Cells

MAGIC-5 cells (CCR5 expressing the HeLa-CD4/long terminal repeat-β-galactosidase cell line; Hachiya et al. 2001) were used as target cells for HIV-1. Cells were maintained in Dulbecco's modified Eagle's medium (ICN, Costa Mesa, CA, USA) supplemented with 10% heat-inactivated fetal bovine serum (Gibco BRL, Grand Island, NY, USA), 100 IU/ml penicillin and 0.1 mg/ml streptomycin.

### Electrodes and the application of electrical potential

The application of electrical potential to HIV-1 was carried out with a three-electrode system using a potentiostat (Toho Technical Research, PS-06, Japan). The working electrode was an optically transparent glass plate (about 50 × 50 mm) sputtered with ITO (Kinoene Optics, Japan; Kumagai et al. 2007; Tominaga et al. 2007). The ITO electrode, with a glass ring (36 mm inner diameter, 15 mm in height) adhered to its surface, was cleaned by sonication in Contaminon N solution (Wako Chemicals, Japan), followed by rinsing with distilled water and autoclaving. A Ag/AgCl (saturated KCl) electrode and a platinum electrode were used as reference and counter electrodes, respectively. All potentials are reported with respect to the Ag/AgCl electrode.

### Adsorption of HIV-1 onto the ITO electrode surface

To adsorb HIV-1 onto the ITO electrode surface, 500 μl of a 0.01% (w/v) of PLL solution (Sigma-Aldrich, P4832; the molecular weight of the polymer was 150,000-300,000 with an estimated1,026-2,052 repeating monomer units) was added and incubated for 5 min before removal by aspiration. The ITO electrode was thoroughly rinsed with sterile water and dried. Then, 1 ml of HIV-1_LAI _or HIV-1_KMT _solution was added to the PLL-coated ITO electrode and incubated for 3 h at 37°C/5% CO_2_.

### Multinuclear activation of a galactosidase indicator (MAGI) assay

HIV-1_LAI _or HIV-1_KMT _that had adsorbed onto the PLL-coated ITO electrode surface was stimulated by a constant d.c. potential of 1.0 V (vs. Ag/AgCl) for 2-10 min. Then, MAGIC-5 cells (15 × 10^4 ^cells/dish) were seeded onto the electrically stimulated HIV-1_LAI_, and cultured for three days at 37°C/5% CO_2_. After removing the supernatant, the HIV-1-infected cells were fixed and stained according to a previously described MAGI assay (Kimpton and Emerman, 1992; Kumagai et al. 2007).

### HIV-1 p24 antigen assay

HIV-1_LAI _(1 ml) solution was added to the PLL-coated ITO electrode, and incubated for 3 h at 37°C/5% CO_2_. After washing twice with PBS, HIV-1_LAI _that had adsorbed onto the ITO electrode surface was dissolved with 1% Triton X. The amount of viral core p24 antigen was measured using an HIV-1 p24 antigen ELISA Kit (ZeptoMetric, NY).

### Measuring number of damaged cells

To calculate number of damaged cells among electrically stimulated cells, cells were stained with 0.4% trypan blue dye for 5 min. After the dye removal, the cells were washed three times with phosphate-buffered saline (PBS; pH7.2). The rate of cell damage was deduced after counting both stained (damaged) and unstained (undamaged) cells under a microscope.

### Cell proliferation assay

To measure cell proliferation, a Cell Counting Kit (CCK; Dojindo, Kumamoto, Japan) was used. MAGIC-5 cells (15 × 10^4 ^cells/3 ml/dish) were seeded onto the PLL-coated ITO electrode and were cultured for 3 h at 37°C. After application of a 1.0-V potential for 2-7.5 min, the cells were cultured for three days in a 5% CO_2 _incubator at 37°C. CCK solution (300 μl) was added to each ITO electrode, and incubated for 40 min 37°C. Then, 100 μl of the supernatant was removed from each ITO electrode and placed in three wells of a 96-flat well plate (Iwaki, Tokyo, Japan), and the absorbance was measured immediately at 450 nm using a microplate reader.

### Apoptosis assay

Electrically stimulated MAGIC-5 cells were fixed with 4% formalin neutral buffer solution for 10 min at room temperature. Fixed cells were then assessed for apoptosis using an Apoptosis *In Situ *Detection Kit (Wako Chemicals). This assay is based on the TdT-mediated dUTP nick end labeling method (TUNEL method).

## Results

### HIV-1 adsorption onto the ITO electrode

The amount of HIV-1 adsorption onto the PLL-coated ITO electrode surface was examined as a preliminary experiment. HIV-1_LAI _(p24 antigen level, 211 ng/ml) was added to four PLL-coated ITO electrodes and four PLL-uncoated ITO electrodes, and incubated for 3 h at 37°C. The mean and standard deviation (SD) of HIV-1_LAI _adsorbed onto four PLL-coated ITO electrode surface was 14.1 ± 0.6 ng/ml, and the rate of absorption was about 6.7%. When the electrode was not coated with PLL, HIV-1_LAI _did not adsorb onto the electrode surface.

### HIV-1 infectivity after electrical stimulation

**Figure 1:**
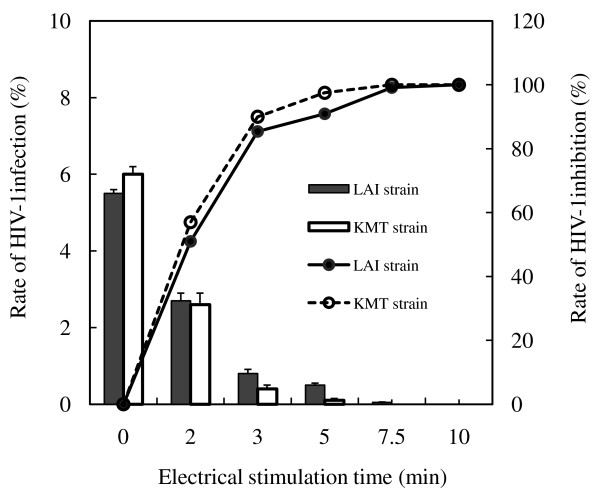
**Effect of electrical stimulation on HIV-1 infectivity**. HIV-1_LAI _or HIV-1_KMT _was incubated for 3 h at 37°C on PLL-coated ITO electrodes and then stimulated by a constant d.c. potential of 1.0 V (vs. Ag/AgCl) for 2 to 10 min. MAGIC-5 cells were then seeded onto the electrically stimulated virus. After culturing for 3 days at 37°C, HIV-1-infected cells were examined using a MAGI assay. More than 3,000 cells were counted under a microscope. The rates of HIV-1_LAI _and HIV-1_KMT _infection were defined as the number of stained cells divided by the total number of cells, as shown in the bar graph. The rates of HIV-1_LAI _and HIV-1_KMT _inhibition were derived from the infection rate of electrically stimulated and unstimulated virus, as shown in the polygonal line graph. Data represent the geometric mean ± standard deviation of duplicate determinations.

HIV-1_LAI _or HIV-1_KMT _were incubated for 3 h on the PLL-coated ITO electrode, which was stimulated by a constant d.c. potential of 1.0 V (vs. Ag/AgCl) for different time periods, ranging from 2-10 min. As shown in Figure 1, the rates of HIV-1_LAI _and HIV-1_KMT _infection progressively decreased with the duration of electrical stimulation, and both types of HIV-1 infection were virtually undetectable after 7.5 min of electrical stimulation.

The rates of HIV-1_LAI _and HIV-1_KMT _inhibition were obtained from the infection rate of electrically stimulated and unstimulated HIV-1, respectively. After application of an electrical potential at 1.0 V (vs. Ag/AgCl) for 2, 3 and 5 min, the rate of HIV-1_LAI _inhibition was approximately 51%, 85% and 91%, and the rate of HIV-1_KMT _inhibition was approximately 57%, 90% and 98%, respectively.

### Cell damage induced by electrical stimulation

**Figure 2:**
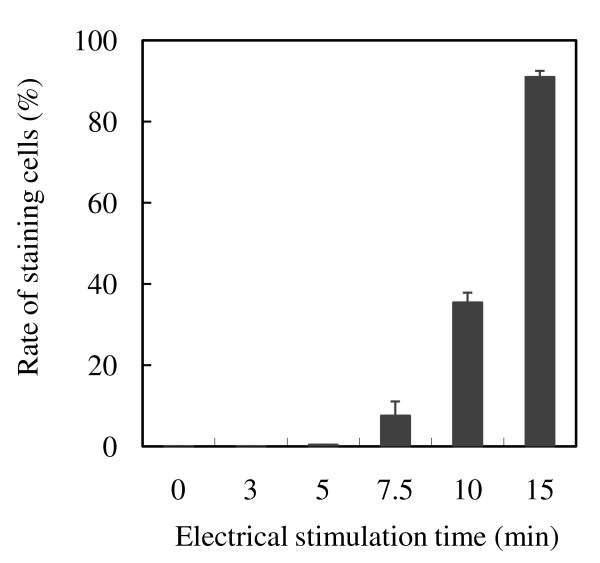
**Rate of damage of MAGIC-5 cells in response to electrical stimulation**. MAGIC-5 cells were cultured for 3 h at 37°C on PLL-coated ITO electrodes and then stimulated by a d.c. potential of 1.0 V (vs. Ag/AgCl) for 3 to 15 min. The cells were stained with 0.4% trypan blue dye for 5 min. More than 1,000 cells were counted under a microscope. Data represent the geometric mean ± standard deviation of duplicate determinations.

MAGIC-5 cells cultured for 3 h on the PLL-coated ITO electrode were stimulated by a constant d.c. potential of 1.0 V (vs. Ag/AgCl) for different time periods, ranging from 3 to 15 min. As shown in Figure 2, the rate of damage of MAGIC-5 cells progressively increased with the duration of electrical stimulation. After electrical stimulation for 3 and 5 min, the cells were barely damaged. However, about 91% of cells were damaged after 15 min stimulation.

### Cell proliferation after electrical stimulation

**Figure 3:**
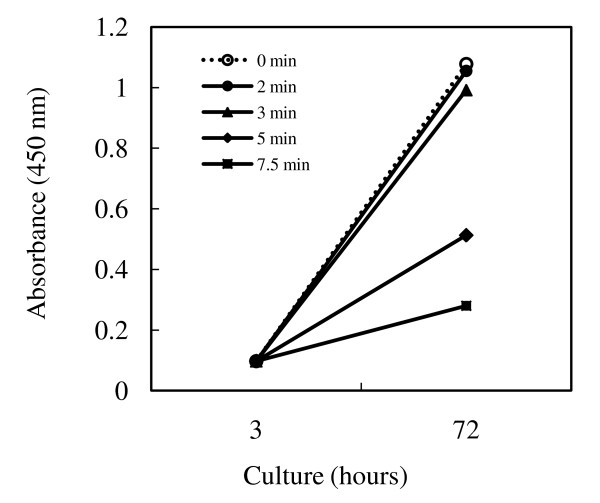
**Proliferation of MAGIC-5 cells in response to electrical stimulation**. MAGIC-5 cells were cultured for 3 h at 37°C on PLL-coated ITO electrodes. The cells were stimulated by a d.c. potential of 1.0 V (vs. Ag/AgCl) for different time periods (2, 3, 5 and 7.5 min) and then cultured for 3 days. Proliferation of cells was measured using a Cell Counting Kit. Data represent the geometric means of triplicate determinations.

Proliferation of MAGIC-5 cells after application of a 1.0 V (vs. Ag/AgCl) electrical potential is shown in Figure 3. Cell proliferation was unchanged in the presence of electrical stimulation at 1.0 V for 3 min, whereas proliferation of the cells was markedly decreased in the presence of electrical stimulation at 1.0 V for 5 min.

### Apoptotic cells after electrical stimulation

**Figure 4:**
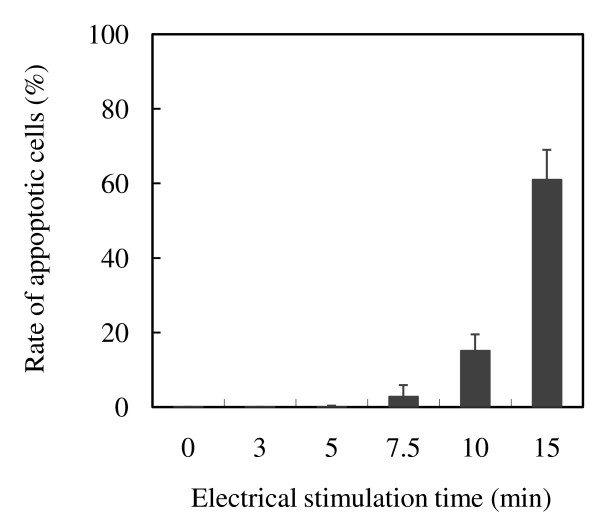
**Apoptotic rate of MAGIC-5 cells in response to electrical stimulation**. MAGIC-5 cells were cultured for 3 h at 37°C on PLL-coated ITO electrodes and then stimulated by a d.c. potential of 1.0 V (vs. Ag/AgCl) for 3 to 15 min. Apoptotic cells were measured using an Apoptosis *In Situ *Detection Kit. More than 1,000 cells were counted under a microscope. Data represent the geometric means of duplicate determinations.

The apoptotic rate of MAGIC-5 cells after application of a 1.0 V (vs. Ag/AgCl) electrical potential is shown in Figure 4. After electrical stimulation at 1.0 V for 5, 7.5 and 15 min, the apoptotic rate of MAGIC-5 cells was about 0.1, 2.8 and 61%, respectively. These rates were lower than the rates of damage of cells stimulated under the same conditions.

## Discussion

A previous study reported that a lowering of cell membrane fluidity was caused not only by electrical stimulation (Kojima et al. 1991), but also by certain medicines and by changes in temperature. Another study demonstrated that use of the local anesthetic xylocaine affected the fluidity of the cell plasma membrane, in turn affecting HIV-1 infectivity (Harada et al. 2005). We predicted that the low potential sensitivity of HIV-1 would be higher than that of cells, because the fluidity of the viral envelope is lower than that of the plasma membrane (Harada et al. 2005).

To stimulate HIV-1 with a low potential, it is first necessary for the virus to be adsorbed onto an ITO electrode. However, HIV-1 does not possess the adhesion plaques exhibited by adherent cells, so was unable to adsorb onto the ITO electrode. Therefore, in our previous report (Kumagai et al. 2007), the effects of electrical stimulation on HIV-1_LAI _were indirectly examined. The research was carried out as follows: HIV-1_LAI _was adsorbed onto MAGIC-5 cells cultured on the ITO electrode, and then the HIV-1_LAI_-adsorbed cells were stimulated by a constant d.c. potential of 1.0 V (vs. Ag/AgCl). When the HIV-1_LAI_-adsorbed cells were stimulated at this potential for 5 min, infection was inhibited by about 37%, but the rate of damage of the HIV-1_LAI_-adsorbed cells was about 1%. After application of a potential of 1.0 V for 5 min, the mean fluorescence intensities of highly ROS and nitric oxide in the HIV-1_NL43-Luc_-adsorbed cells were significantly increased compared with those of unstimulated cells. These results suggested that the membrane of cells and virus envelopes were changed by electrical stimulation. As a result, the entry of viruses into cells might be blocked. However, it could not be completely ruled out that a few HIV-1 that had already entered cells at the point of stimulation might have been exposed to the effects of electrical stimulation. ROS that exhibit anti-viral activity (Corasaniti et al. 1995) might be involved in this process.

In the current study, HIV-1 and MAGIC-5 cells were directly stimulated with a constant d.c. potential of 1.0 V (vs. Ag/AgCl). The sensitivities of HIV-1 and the cells to electrical stimulation were then examined. PLL was used as an attachment factor for HIV-1 onto the ITO electrode, and about 7% of HIV-1_LAI _was adsorbed onto the electrode surface by coating it with PLL. After adsorption, HIV-1 was directly stimulated with a potential of 1.0 V, and then MAGIC-5 cells were seeded onto HIV-1. After culturing the cells at 37°C for three days, the rate of HIV-1 infection was examined. MAGIC-5 cells were also used as the target cells of HIV-1, as they are easily infected with HIV and their cell morphology is easy to observe.

By directly stimulating HIV-1_LAI _or HIV-1_KMT _adsorbed onto the PLL-coated ITO electrode with the potential of 1.0 V (vs. Ag/AgCl), the infectivity of both types of HIV-1 was remarkably inhibited. For example, HIV-1 (HIV-1_LAI _or HIV-1_KMT_) infection was inhibited by about 90% by electrical stimulation of 1.0 V for 3 min. By application of the potential for 5 min, the infection inhibition rate of HIV-1_LAI _(about 91%) was more than twice that of HIV-1_LAI_-adsorbed MAGIC-5 cells. The MAGI assay is a method for determining inactivation of the β-galactosidase gene when HIV-1 is integrated into the DNA of MAGIC-5 cells (Kimpton et al. 1992). The results of this assay indicated that lowering HIV-1 infectivity by electrical stimulation prevented HIV-1 from integrating into the DNA of the host cell. However, it remains unclear which part of this process, from adsorption of HIV-1 to cells to DNA integration, was damaged by electrical stimulation. With the currently available methodology, it is not possible to clarify this point. With future improvements in the ITO electrode, it may be possible to examine this is more detail.

Our results also demonstrated that there were no changes in the rate of cell damage, the apoptotic rate or the rate of cell proliferation in MAGIC-5 cells after electrical stimulation of 1.0 V (vs. Ag/AgCl) for 3 min, compared with unstimulated cells. After application of the potential for 5 min, damaged cells and apoptotic cells were virtually undetectable, however, the proliferation of cells also decreased by about 50%, so low levels of DNA damage not detected by the apoptosis assay might have influenced the proliferation of cells. Taken together, these findings suggested that HIV-1 was significantly more susceptible to the electrical potential of 1.0 V (vs. Ag/AgCl) than cells.

In conclusions, we have shown that HIV-1 is significantly damaged by a d.c. potential of 1.0 V compared with cells. This remarkable difference in sensitivity between HIV-1 and cells to electrical stimulation could be useful not only for the elucidation of HIV control mechanisms but also for the development of novel therapies for HIV-1.

## Competing interests

The authors declare that they have no competing interests.
